# Induced pluripotent stem cell‐derived conditional medium promotes Leydig cell anti‐apoptosis and proliferation via autophagy and Wnt/β‐catenin pathway

**DOI:** 10.1111/jcmm.13641

**Published:** 2018-04-18

**Authors:** Xiaoling Guo, Yong Chen, Tingting Hong, Xianwu Chen, Yue Duan, Chao Li, Renshan Ge

**Affiliations:** ^1^ Center of Scientific Research The Second Affiliated Hospital and Yuying Children's Hospital of Wenzhou Medical University Wenzhou Zhejiang China; ^2^ Department of Anesthesiology The Second Affiliated Hospital and Yuying Children's Hospital of Wenzhou Medical University Wenzhou Zhejiang China

**Keywords:** apoptosis, immature Leydig cells, induced pluripotent stem cell‐derived conditional medium, pathway, proliferation

## Abstract

Leydig cell transplantation is a better alternative in the treatment of androgen‐deficient males. The main purpose of this study was to investigate the effects of induced pluripotent stem cell‐derived conditioned medium (iPS‐CM) on the anti‐apoptosis, proliferation and function of immature Leydig cells (ILCs), and illuminate the underlying mechanisms. ILCs were exposed to 200 μmol/L hydrogen peroxide (H_2_O_2_) for 24 hours with or without iPS‐CM treatments. Cell apoptosis was detected by flow cytometric analysis. Cell proliferation was assessed using cell cycle assays and EdU staining. The steroidogenic enzyme expressions were quantified with Western blotting. The results showed that iPS‐CM significantly reduced H_2_O_2_‐induced ILC apoptosis through down‐regulation of autophagic and apoptotic proteins LC3‐I/II, Beclin‐1, P62, P53 and BAX as well as up‐regulation of BCL‐2, which could be inhibited by LY294002 (25 μmol/L). iPS‐CM could also promote ILC proliferation through up‐regulation of β‐catenin and its target proteins cyclin D1, c‐Myc and survivin, but was inhibited by XAV939 (10 μmol/L). The level of bFGF in iPS‐CM was higher than that of DMEM‐LG. Exogenous bFGF (20 ng/mL) or Wnt signalling agonist lithium chloride (LiCl) (20 mmol/L) added into DMEM‐LG could achieve the similar effects of iPS‐CM. Meanwhile, iPS‐CM could improve the medium testosterone levels and up‐regulation of LHCGR, SCARB1, STAR, CYP11A1, HSD3B1, CYP17A1, HSD17B3 and SF‐1 in H_2_O_2_‐induced ILCs. In conclusion, iPS‐CM could reduce H_2_O_2_‐induced ILC apoptosis through the activation of autophagy, promote proliferation through up‐regulation of Wnt/β‐catenin pathway and enhance testosterone production through increasing steroidogenic enzyme expressions, which might be used in regenerative medicine for future.

## 1 INTRODUCTION

Leydig cells, distributing in clusters between the seminiferous tubules in the testis, are responsible for androgen production in the male.^1^, ^2^ Testosterone, referred to as the male hormone, plays a critical role in maintaining sexual function, muscle bulk and bone health.^3^ Testosterone synthesis in Leydig cells depends on the luteinizing hormone (LH) secreted by the pituitary grand.^4^ LH binds LH receptors (LHCGR, encoded by *Lhcgr*) on the Leydig cell plasma membrane and then leads to intracellular cyclic adenosine monophosphate (cAMP) cascade,^5^ which further results in the rapid transport of cholesterol from the outer to the inner mitochondrial membrane, mediated by lipoprotein receptor (SCARB1, encoded *Scarb1*) and steroidogenic acute regulatory protein (STAR, encoded by *Star*). Subsequently, testosterone is synthesized through a series of steroidogenic enzymes: the cytochrome P450 cholesterol side‐chain cleavage enzyme (CYP11A1, encoded by *Cyp11a1*), 3β‐hydroxysteroid dehydrogenase (HSD3B1, encoded by *Hsd3b1*), cytochrome P450 17α‐hydroxylase (CYP17A1, encoded by *Cyp17a1*) and 17β‐hydroxysteroid dehydrogenase 3 (HSD17B3, encoded by *Hsd17b3*),^6^ while another critical factor for Leydig cells is the steroidogenic factor 1 (SF‐1, or named NR5A1), which is essential for the commitment of stem Leydig cells to testosterone‐producing Leydig cells.^7^ It is well known that SF‐1 is an essential factor for Leydig cell development and survival.^8^ Overexpression of SF‐1 has been shown to be capable of promoting ESCs into Leydig‐like cells.^9^

Induced pluripotent stem cells (iPSCs) are a type of pluripotent stem cells that can be generated directly from adult cells.^10^, ^11^, ^12^ iPSCs share the features of ESCs that are capable of self‐renewal and differentiation into three germ layers.^13^ iPSCs can address immune rejection and ethical issues of the autologous cell transplantation. They also offer an attractive approach to disease model, pharmaceutical screening, toxicology research and so on.^14^, ^15^ Conditioned medium can affect cell functions via biologically active components. It was reported that cytokines such as basic fibroblast growth factor (bFGF), nerve growth factor (NGF), stem cell factor (SCF), hepatocyte growth factors (HGF), vascular endothelial growth factor (VEGF), insulin‐like growth factor (IGF‐1) and brain derived neurotrophic factor (BDNF), which were secreted in the medium by cultured stem cells, could encourage the growth of cells.^16^, ^17^, ^18^ Li et al^11^ reported that induced pluripotent stem cell‐derived conditioned medium (iPS‐CM) potentially restored the bronchial microstructure in acute lung injury (ALI). Zhang et al^19^ mentioned that iPS‐CM contributed to recovery from the effect of endotoxin‐induced ALI in mice. The stimulating proliferation and anti‐apoptosis from iPS‐CM were the result of cytokines secretion, which were generally safe and would not produce tumours.^19^, ^20^, ^21^

The Wnt/β‐catenin signalling pathway is the classic pathway involved in cell proliferation.^22^ Basic fibroblast growth factor (bFGF) could promote the proliferation and migration of fibroblasts through the activation of Wnt/β‐catenin signalling pathway.^23^ Autophagy has been known as a conserved catabolism to sustain cellular homeostasis.^24^, ^25^ It plays critical roles in eliminating dysfunctional or surplus proteins and damaged intracellular organelles.^26^ A major event in autophagosome formation is to generate LC3‐I, which is then conjugated to phosphatidylethanolamine, generating membrane‐bound LC3‐II.^27^, ^28^ Beclin‐1 is a key autophagic protein regulating autophagosome formation.^29^, ^30^ Meanwhile, P62 is a specific autophagic substrate protein and the hallmark representing autophagic flu.^30^ It is known that the activation of autophagy can protect cells against apoptosis and inflammation.^31^, ^32^

In this research, we would investigate the effects of iPS‐CM on the proliferation and the H_2_O_2_‐induced apoptosis of immature Leydig cells (ILCs), and evaluate the influences of iPS‐CM on testosterone production of ILCs. Moreover, the potential mechanisms underlying the effects of iPS‐CM would be explored. This study was to develop a new way to improve the activities and function of ILCs used for future clinical cell transplantation.

## 2 MATERIALS AND METHODS

### 2.1 Chemicals and kits

Etiocholanolone, nicotinamide adenine dinucleotide (NAD^+^), collagenase, DNase, Percoll, hydrogen peroxide (H_2_O_2_), Y‐27632, Matrigel, M‐199 buffer, Triton X‐100, DAPI, XAV939, LY294002 and lithium chloride (LiCl) were purchased from Sigma‐Aldrich (St. Louis, MO, USA). Low‐glucose Dulbecco's modified Eagle's medium, fatal bovine serum (FBS), 1% penicillin and streptomycin, and 0.05% trypsin‐EDTA were purchase from Gibco Company (NY, USA). Annexin V‐FITC/PI apoptosis detection kit, 5, 59, 6, 69‐tetrachloro‐1, 19, 3, 39‐tetraethylbenzimi‐dazolylcarbocyanine iodide (JC‐1) assay kit was purchased from Nanjing KeyGEN Biotech (Nanjing, China). 2′7′‐Dichlorofluorescin diacetate (DCFH‐DA) assay kit was purchased from Qcbio Science & Technologies Co., Ltd. (Shanghai, China).

### 2.2 Animals

The Sprague‐Dawley rats were provided by Laboratory Animal Center, Wenzhou Medical University, Wenzhou, China. They were raised in a 12‐hour dark/light cycle temperature at 23 ± 2°C, and relative humidity of 45% to 55%. Water and food were accessed ad libitum. This study was approved by the Wenzhou Medical University's Animal Care and Use Committee, and was performed in accordance with the Guide for the Care and Use of Laboratory Animals.

### 2.3 Immature Leydig cells isolation

Eighteen 35‐day‐old male Sprague‐Dawley rats were killed in CO_2_ tank for the isolation of immature Leydig cells (ILCs). ILCs express all androgen biosynthetic enzymes^33^ and are capable of proliferation.^34^ The isolation of rat ILCs was performed as previously described.^33^ Briefly, the testes were removed, perfused with collagenase via the testicular artery and digested with M‐199 buffer containing collagenase (0.25 mg/mL) and DNase (0.25 mg/mL) for 15 minutes. Then, the cell suspension was filtered through 100‐μm nylon mesh (BD, CA, USA) and the cells were separated by the Percoll gradient. The cells with the density of 1.07‐1.088 g/mL were collected. The purity of ILCs was evaluated by immunohistochemical staining HSD3B1, the biomarker of ILCs, as previously described.^35^ The HSD3B1 staining solution contained with 0.4 mmol/L etiocholanolone as the steroid substrate and NAD^+^ as a cofactor.^35^ The purity of ILCs was more than 95%.

### 2.4 Culture of ILCs

The isolated ILCs were directly seeded into wells in the 24‐well culture plates with the density of 2 × 10^4^ cells/well and incubated at a 37°C, 5% CO_2_ incubator. The culture medium (DMEM‐LG) contains low‐glucose Dulbecco's modified Eagle's medium, 10% fatal bovine serum (FBS), and 1% penicillin and streptomycin as control.

### 2.5 The culture of iPSCs and preparation of iPSC supernatant

iPSCs were cultured as previously described.^36^ In brief, culture plates were coated with 1% (v/v) Matrigel for 0.5 hours in advance, and then, iPSCs were cultured in mTeSR1 medium (StemCell Technologies Inc., Canada) at a 37°C, 5% CO_2_ incubator. These cells would be passaged every 6 days using 0.05% trypsin‐EDTA. Y‐27632 (10 mmol/L) as ROCK inhibitor was added into the plates on the first day after passage. The supernatant of iPSC was derived from the mTeSR1 medium cultured with iPSCs for 24 hours. The supernatant was filtered (0.22 μm) to remove dead cells and then stored at −80°C for at least 2 weeks. iPS‐CM was DMEM‐LG mixed with iPSC supernatant at the ratio of 2:1.

### 2.6 CCK‐8 assay

CCK‐8 was used to detect the cell viability of ILCs with different treatments. Briefly, isolated ILCs were seeded in the 96‐well plates at the density of 1 × 10^4^ cells/well. Then, cells were cultured in different mediums and incubated at a 37°C, 5% CO_2_ incubator for 48 hours. Subsequently, the each well was supplemented with 10 μL CCK‐8 (BestBio, Shanghai, China) solution and incubated at 37°C for 4 hours. At last, the absorbance at 450 nm was measured with a microplate reader (Thermo, MA, USA).

### 2.7 Cell cycle assay

Freshly isolated ILCs were seeded in the 12‐well plates at the density of 5 × 10^5^ cells/well and incubated for 24 hours. Then, the medium was changed into DMEM‐LG or iPS‐CM with 200 μmol/L H_2_O_2_. Cells were cultured for 48 hours. Then, cells were harvested and fixed with 75% cold ethanol overnight at 4°C. The fixed cells were washed once with phosphate‐buffered saline (PBS) and stained darkly with propidium iodide (PI) for 30 minutes at room temperature. The stained cells were analysed by flow cytometer (BD FACSAria, San Diego, CA, USA).

### 2.8 Annexin V and PI assay

ILCs were planted into the 12‐well plates with the density of 5 × 10^5^ cells/well and incubated for 24 hours. Then, the medium was changed into DMEM‐LG or iPS‐CM with 200 μmol/L H_2_O_2_. Cells were cultured for 48 hours. To evaluate early and lately apoptotic activity, an Annexin V‐FITC/PI apoptosis detection kit was used as the manufacturer's instructions. Cells were harvested and washed with cold PBS and then were resuspended in 200 μL the Annexin V‐binding buffer. After cells were stained with 5 μL of FITC‐labelled Annexin V and 5 μL of PI, they were instantly measured using flow cytometer.

### 2.9 Measurement of mitochondrial membrane potential (▵Ψm)

▵Ψm was evaluated with JC‐1 assay kit. Approximately 5 × 10^5^ cells/well‐isolated ILCs were plated into the 12‐well plates and incubated for 24 hours. Then, the medium was changed into DMEM‐LG or iPS‐CM with 200 μmol/L H_2_O_2_. Cells were cultured for 48 hours. Subsequently, culture medium was removed and cells were washed in cold PBS twice. Cells were incubated for 30 minutes at 37°C with 500 μL JC‐1 (5 mmol/L). Cells were collected by centrifugation at 500 g for 5 minutes and washed twice again with 1× incubation buffer. Cells were then resuspended with 500 μL 1× incubation buffer. Red and green fluorescence emissions were analysed by flow cytometer using an excitation wavelength of 488 nm and emission wavelengths of 530 nm (green fluorescence)/585 nm (red fluorescence). Mitochondrial depolarization was assessed by a decrease in the intensity ratio of the red/green fluorescence.

### 2.10 Measurement of cellular H_2_O_2_‐induced reactive oxygen species

Reactive oxygen species (ROS) production was measured with the fluorescence dye DCFH‐DA assay kit. Briefly, 5 × 10^5^ cells/well‐isolated ILCs were plated into the 12‐well plates and incubated for 24 hours. Then, the medium was changed into DMEM‐LG or iPS‐CM with 200 μmol/L H_2_O_2_. Cells were cultured for 48 hours. Thereafter, cells were harvested and suspended with 200 μL DCFH‐DA for 20 minutes at 37°C in the dark. Cells were washed twice with PBS, and fluorescence intensity determined by flow cytometer was used to measure ROS.

### 2.11 Cell treatments

For exploration of the underlying mechanisms, 1 × 10^6^ cells/well‐isolated ILCs were plated into the 6‐well plates and incubated for 24 hours. For exploration the apoptosis mechanism, the mediums were changed into DMEM‐LG (control) or iPS‐CM with 200 μmol/L H_2_O_2_ to establish control+H_2_O_2_ group or iPS‐CM+H_2_O_2_ group. Exogenous bFGF (20 ng/mL) was added into DMEM‐LG+H_2_O_2_ to obtain the control+H_2_O_2_+bFGF group. LY294002 (LY, 25 μmol/L) were added into iPS‐CM+H_2_O_2_ to get iPS‐CM+H_2_O_2_+LY group. Then, cells were cultured for another 48 hours and harvested for Western blotting. For exploration of the proliferation mechanism, the mediums were changed into DMEM‐LG (control) or iPS‐CM to establish control group or iPS‐CM group. XAV939 (XAV, 10 μmol/L) was added into iPS‐CM to get iPS‐CM+XAV group. Exogenous bFGF (20 ng/mL) was added into DMEM‐LG to get control+bFGF group. LiCl (20 mmol/L) was added into DMEM‐LG to get control+LiCl group. Then, cells were cultured for another 48 hours and harvested for Western blotting.

### 2.12 Western blotting

Cells were washed twice with cold PBS and then were lysed in the radio immunoprecipitation assay buffer (Bocai Biotechnology, Shanghai, China) supplemented with a protease inhibitor (Amyjet Scientific Inc, Wuhan, China). Lysate was centrifuged at 12 000 *g* for 15 minutes at 4°C. The protein concentrations in the supernatants were measured using the BCA assay kit (Takara, Japan) as the manufacturer's instructions. Sample proteins (50 μg) were subjected to 10% polyacrylamide gel containing sodium dodecyl and then transferred into the polyvinylidene fluoride membrane. After being blocked with 5% free‐fat milk in Tween 20‐containing Tris‐buffered saline for 2 hours at 4°C, the membranes were incubated with primary antibodies over night at 4°C (listed in Table 1). Then, membranes were washed with Tween 20‐containing Tris‐buffered saline for five times and incubated with horseradish peroxidase‐conjugated secondary antibody (1:5000, Bioword, MN, USA) for 1 hour at room temperature and then were washed with the buffer for three times again. The protein bands were visualized with enhanced chemiluminescence (Pierce Chemical Co, IL, USA). The intensities of proteins were quantified using Image J software.

**Table 1:** Antibodies

Antibody	Species	Vendor (city, state, catalogue)	WB
β‐Actin	Rabbit	Cell Signaling Technology (Danvers, MA, 12620S)	1:1000
LHCGR	Goat	Santa Cruz (Santa Cruz, CA, sc‐26343)	1:1000
SCARB1	Rabbit	Abcam (San Francisco, CA, ab217318)	1:1000
STAR	Rabbit	Abcam (San Francisco, CA, ab133657)	1:1000
CYP11A1	Rabbit	Santa Cruz (Santa Cruz, CA, sc‐18043)	1:1000
HSD3B1	Rabbit	Abcam (San Francisco, CA, ab65156)	1:2000
CYP17A1	Rabbit	Santa Cruz (Santa Cruz, CA, sc‐66850)	1:1000
HSD17B3	Rabbit	Abcam (San Francisco, CA, Ab70088)	1:2000
SF‐1	Rabbit	Santa Cruz (Santa Cruz, CA, sc‐10976)	1:1000
BAX	Rabbit	Cell Signaling technology (Danvers, MA, 2774S)	1:1000
BCL2	Rabbit	Cell Signaling technology (Danvers, MA, 4223S)	1:1000
β‐catenin	Rabbit	Cell Signaling technology (Danvers, MA, 8480)	1:1000
Cyclin D1	Mouse	Santa Cruz (Santa Cruz, CA, sc‐8396)	1:2000
c‐Myc	Mouse	Santa Cruz (Santa Cruz, CA, sc‐40)	1:2000
Survivin	Mouse	Santa Cruz (Santa Cruz, CA, sc‐73082)	1:2000
LC3‐I/II	Rabbit	Cell Signaling technology (Danvers, MA, 12741S)	1:1000
Beclin‐1	Rabbit	Cell Signaling technology (Danvers, MA, 3495S)	1:1000
P62	Rabbit	Cell Signaling technology (Danvers, MA, 39749S)	1:1000
P53	Mouse	Cell Signaling technology (Danvers, MA, 2524S)	1:1000

### 2.13 Testosterone measurement by radioimmunoassay

Medium testosterone concentrations were measured with a tritium‐based radioimmunoassay using antitestosterone antibody as previously described.^37^ Standards ranging between 10 and 2000 pg/mL testosterone were prepared in triplicate. Standards and samples were incubated with tracer, and charcoal‐dextran suspension was used to separate the bound and free steroids. The bound steroids were mixed with a scintillation buffer and counted in a β scintillation counter (PE, CA, USA). The minimum detectable concentration for testosterone was 5 pg/mL. Quality control samples contain 100 pg/mL testosterone. The intra‐assay and interassay coefficients of variation were within 10%.

### 2.14 EdU staining assays

EdU staining was conducted with a Click‐iT^®^ EdU imaging kit (Invitrogen, CA, USA) as per manufacturer's instruction. Briefly, isolated ILCs were seeded in the 12‐well plates at the density of 5 × 10^5^ cells/well and incubated for 24 hours. Then, cells were incubated with EdU at a final concentration of 20 μmol/L at 37°C incubator for 2 hours. Cells were harvested by digestion and centrifugation, and were fixed with 4% paraformaldehyde for 15 minutes and permeabilized using Triton X‐100 solution for 30 minutes at room temperature darkly. Then, cells were centrifuged and resuspended in 0.5 mL of PBS. Fixed cells were stained with the Click‐iT™ reaction mixture. Then, cell nucleus was stained with 1 μg/mL DAPI at room temperature for 30 minutes darkly. Lastly, the stained cells were observed at an inverted fluorescence microscopy and were analysed using flow cytometry.

### 2.15 ELISA

The samples were treated with enzyme‐linked immunosorbent assay (ELISA) kit according to the manufacturer's instruction (Chemicon, CA, USA). Briefly, 50 μL assay diluent and 200 μL samples were added to pre‐coated wells of 96‐well plates. The plates were incubated at room temperature for 2 hours and washed five times with washing buffer. 100 μL peroxidase‐conjugated IgG anti‐bFGF of solution was added to each well at room temperature for 2 hours. Then, plates were washed five times with washing buffer. Then 100 μL substrate buffers were added to each well and incubated in dark at room temperature for 30 minutes. The enzyme reaction was stopped by 50 μL stop solution. Optical densities were obtained for the quantification of bFGF levels by a microplate reader at 550 nm with correction wavelength at 450 nm. Data were conducted by GraphPad Prism 5 software.

### 2.16 Statistical analysis

All data are presented as the mean ± standard errors (SE). Statistical significance was analysed using one‐way ANOVA followed by ad hoc Turkey multiple comparisons to the control. Statistical analyses were performed using GraphPad Prism (version 6, GraphPad Software Inc., San Diego, CA, USA). *P <* *.05, **P <* *.01 or ***P <* *.001 were considered statistically significant.

## 3 RESULTS

### 3.1 The optimal iPS‐CM ratio and apoptosis model of H_2_O_2_‐induced immature Leydig cells

**Figure 1:**
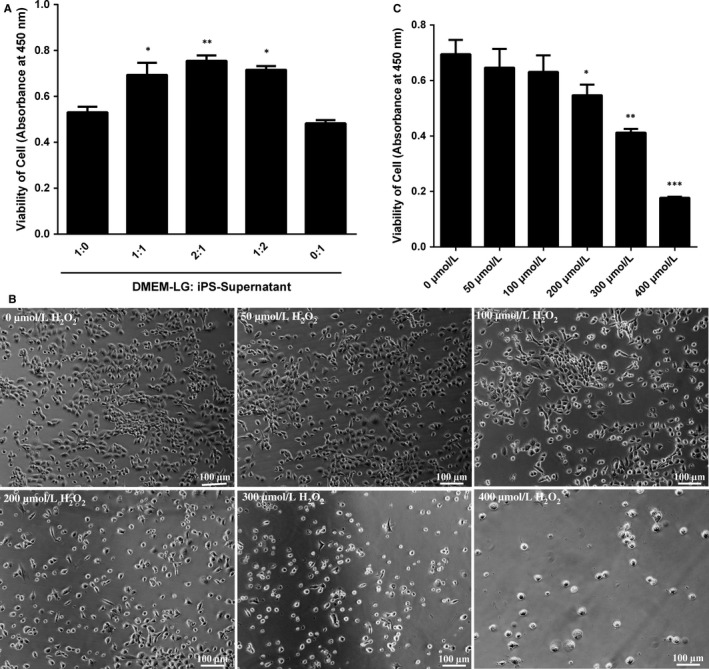
Exploration the optimal proportion of DMEM‐LG/iPS‐supernatant and apoptosis model of H_2_O_2_‐induced immature Leydig cells (ILCs). (A) The proliferation viability of ILCs in different proportion of DMEM‐LG/iPS‐supernatant was analysed by CCK‐8. (B) The bright field of ILCs exposed to various concentrations of H_2_O_2_ under an inverted microscope. (C) The viability effect of iPS‐CM on ILCs exposed to H_2_O_2_ at various doses by CCK‐8 assay. Mean ± SE, n = 5. *P <* *.05, **P <* *.01, ***P <* *.001 designate significant differences when compared to control (1:0 or 0 μmol/L)

The proliferation viability of ILCs was analysed by CCk‐8 to assess the optimal proportion of DMEM‐LG/iPS‐supernatant. ILCs were treated with different proportions of DMEM‐LG/iPS‐supernatant (1:0, 1:1, 2:1, 1:2, 0:1) for 48 hours. The result showed that the proliferation viability of ILCs was the most obvious at the ratio of 2:1 compared with other proportions (Figure 1A). Base on these data, we considered this proportion as iPS‐CM to treat ILCs in following studies.

To establish the optimal apoptosis model, ILCs were exposed to different concentrations of H_2_O_2_ at 0, 50, 100, 200, 300 μmol/L and then were cultured for 24 hours. The death cells were washed away with PBS. The attached cells were imaged using an inverted phase light microscope (Figure 1B). The morphological features of H_2_O_2_‐induced ILCs were examined. The hallmark of apoptosis cells such as cell detachment and cytoplasmic condensation would worsen due to increasing dosage of H_2_O_2_ exposure beginning from the dosage of 200 μmol/L. The result of CCK‐8 assay was consistent with above performance (Figure 1C). These data indicated that H_2_O_2_ at the dose of 200 μmol/L was optimal for the apoptosis model.

### 3.2 Effects of iPS‐CM on the anti‐apoptosis of H_2_O_2_‐induced immature Leydig cells

**Figure 2:**
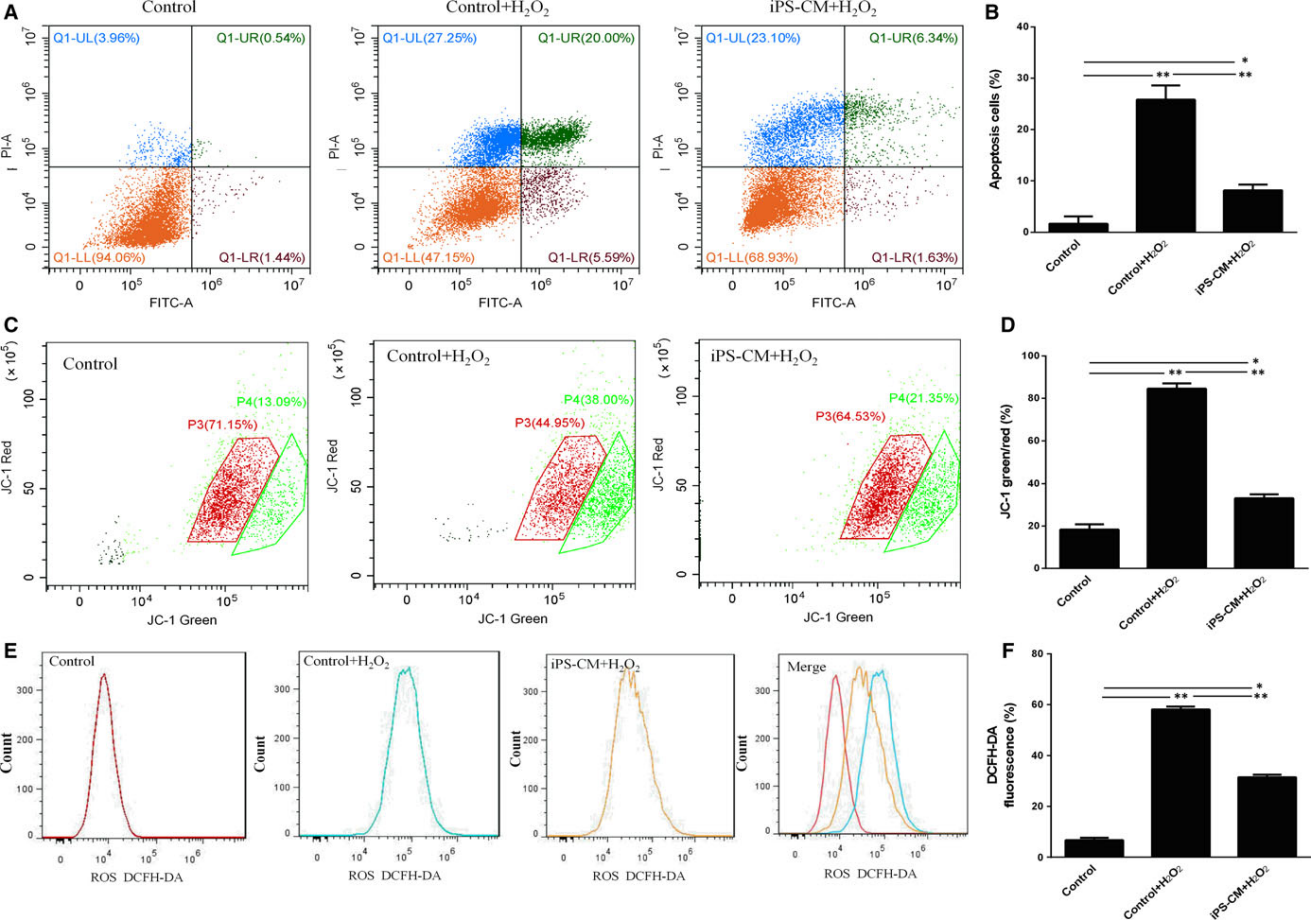
iPS‐CM inhibited the apoptosis of H_2_O_2_‐induced immature Leydig cells (ILCs). (A) Annexin V and propidium iodide (PI) assay was used to analyse the cell apoptosis in different groups. (B) Quantification of the Annexin V and PI assay. (C) Mitochondrial membrane potential (▵Ψm) assay was used to analyse the loss of ▵Ψm of ILCs in different groups. (D) Quantification of ▵Ψm. (E) Induced reactive oxygen species (ROS) assay was used to analyse the generation of ROS in different groups. (F) Quantification of intracellular ROS. Mean ± SE, n = 3. *P <* *.05 (iPS‐CM+H_2_O_2_ vs control), **P <* *.01 (iPS‐CM+H_2_O_2_ or control vs control+H_2_O_2_) designate significant differences

Annexin V and PI assay was conducted to assess the effects of iPS‐CM on the apoptosis of H_2_O_2_‐induced ILCs. In the early stage of apoptosis, cells have the intact cell membranes that cannot be stained by PI. However, externalization of phosphatidylserine can be stained by Annexin V. In the late stage of apoptosis, the damaged cell membranes allow Annexin V and PI to enter into cells. Hence, ILCs in early apoptosis were Annexin V positive and PI negative but in late apoptosis were both Annexin V and PI positive. The percentage of apoptotic cells was calculated from the Q1‐LR (early stage of apoptosis) and Q1‐UR (late stage of apoptosis). Annexin V and PI assays showed that iPS‐CM could decrease the apoptosis ratio of H_2_O_2_‐induced ILCs (Figure 2A). The control group (ILCs in DMEM‐LG) almost had no apoptotic cells, with only 1.98 ± 0.15%. The control+H_2_O_2_ group had 25.59 ± 0.37% apoptotic cells, which is much higher than that of control (**P <* *.01). The apoptotic ratio in iPS‐CM+H_2_O_2_ group was 7.97 ± 0.21%, which was higher than that of control group (*P <* *.05) but lower than that of control+H_2_O_2_ group (**P <* *.01) (Figure 2B). This result demonstrated that iPS‐CM could inhibit H_2_O_2_‐induced ILC apoptosis.

As the loss of ▵Ψm was associated with early apoptosis, JC‐1 probe was used to analyse the effects of iPS‐CM on the loss of ▵Ψm in H_2_O_2_‐induced ILCs. The ▵Ψm of living cells is higher than that of apoptotic cells. JC‐1 can specially enter the mitochondria. When the ▵Ψm is high, JC‐1 will form polymer and glow red fluorescent. When the ▵Ψm is low, it will form the monomer and glow green fluorescence. The ratio of JC‐1‐green/red can indirectly reflect the result of apoptosis. Low ratio presents less apoptosis than high ratio. The loss of ▵Ψm in ILCs after H_2_O_2_ treatment increased obviously compared with that of control group (ILCs in DMEM‐LG), and was prevented by iPS‐CM (Figure 2C). The data showed that the ratio of JC‐1‐green/red in control group was 18.39 ± 2.31%, which was less than that of control+H_2_O_2_ group (84.5 ± 4.54%) (**P <* *.01) and iPS‐CM+H_2_O_2_ group (33.08 ± 2.24%) (*P <* *.05). The ratio of JC‐1‐green/red in iPS‐CM+H_2_O_2_ group was also less than that of control+H_2_O_2_ group (**P <* *.01) (Figure 2D). This result showed that iPS‐CM could inhibit the loss of ▵Ψm in H_2_O_2_‐induced ILCs.

DCFH‐DA was used to detect the effects of iPS‐CM on the generation of ROS in H_2_O_2_‐induced ILCs. The generation of ROS in ILCs after H_2_O_2_ treatment increased remarkably compared with that of control group (ILCs in DMEM‐LG), and then, iPS‐CM decreased the ROS generation of H_2_O_2_‐induced ILCs again (Figure 2E). The result showed that the value of DCFH‐DA fluorescence in control group was 7.8 ± 0.48%, which was less than that of control+H_2_O_2_ group (58.2 ± 3.58%) (**P <* *.01) and iPS‐CM+H_2_O_2_ group (31.5 ± 1.48%) (*P <* *.05). Moreover, the value of DCFH‐DA fluorescence in iPS‐CM+H_2_O_2_ group was less than control+H_2_O_2_ group, having approximately a onefold decrease (**P <* *.01) (Figure 2F). The value of DCFH‐DA fluorescence could reflect the generation of ROS. This result documented that iPS‐CM could inhibit the generation of ROS in H_2_O_2_‐induced ILCs.

All the above data showed that iPS‐CM could inhibit the apoptosis, the loss of ▵Ψm and the generation of ROS in H_2_O_2_‐induced ILCs.

### 3.3 Effects of iPS‐CM on the BAX and BCL‐2 expressions of H_2_O_2_‐induced immature Leydig cells

**Figure 3:**
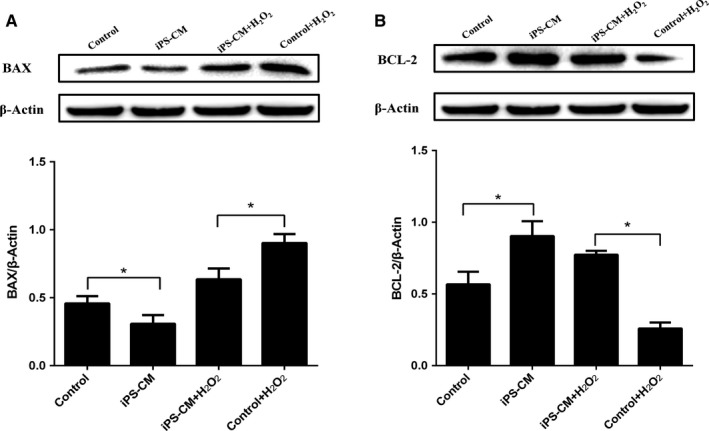
iPS‐CM suppressed BAX up‐regulation and BCl‐2 down‐regulation of H_2_O_2_‐induced immature Leydig cells (ILCs). (A) The protein expression of BAX in different groups. (B) The protein expression of BCL‐2 in different groups. Mean ± SE, n = 3. *P <* *.05 designates significant differences

To explore the potential molecular mechanisms involved in the anti‐apoptosis of iPS‐CM on H_2_O_2_‐induced ILCs, the expression levels of apoptotic signalling molecules in the mitochondria including BAX and BCL‐2 were detected by Western blotting in different groups. The result showed that H_2_O_2_ could apparently up‐regulate the expression of BAX and down‐regulate the expression of BCL‐2. Compared with control group (ILCs in DMEM‐LG), the expression level of BAX in iPS‐CM was less (*P <* *.05). When cells treated with H_2_O_2_, BAX expressions were up‐regulated in control+H_2_O_2_ and iPS‐CM+H_2_O_2_ groups, and the level in iPS‐CM+H_2_O_2_ group was less than that of control+H_2_O_2_ group (*P <* *.05) (Figure 3A). The tendency of BCL‐2 expression was almost opposite to the BAX expression. The BCL‐2 expression level in iPS‐CM group was more than control group (*P <* *.05), and H_2_O_2_ could significantly inhibit the expression of BCL‐2 in control+H_2_O_2_ and iPS‐CM+H_2_O_2_ groups. In addition, the level of BCL‐2 in iPS‐CM+H_2_O_2_ group was higher than that of control+H_2_O_2_ group (*P <* *.05) (Figure 3B). These results showed that iPS‐CM could suppress the up‐regulation of BAX and down‐regulation of BCl‐2 in H_2_O_2_‐induced ILCs.

### 3.4 Effects of iPS‐CM on the proliferation of H_2_O_2_‐induced immature Leydig cells

**Figure 4:**
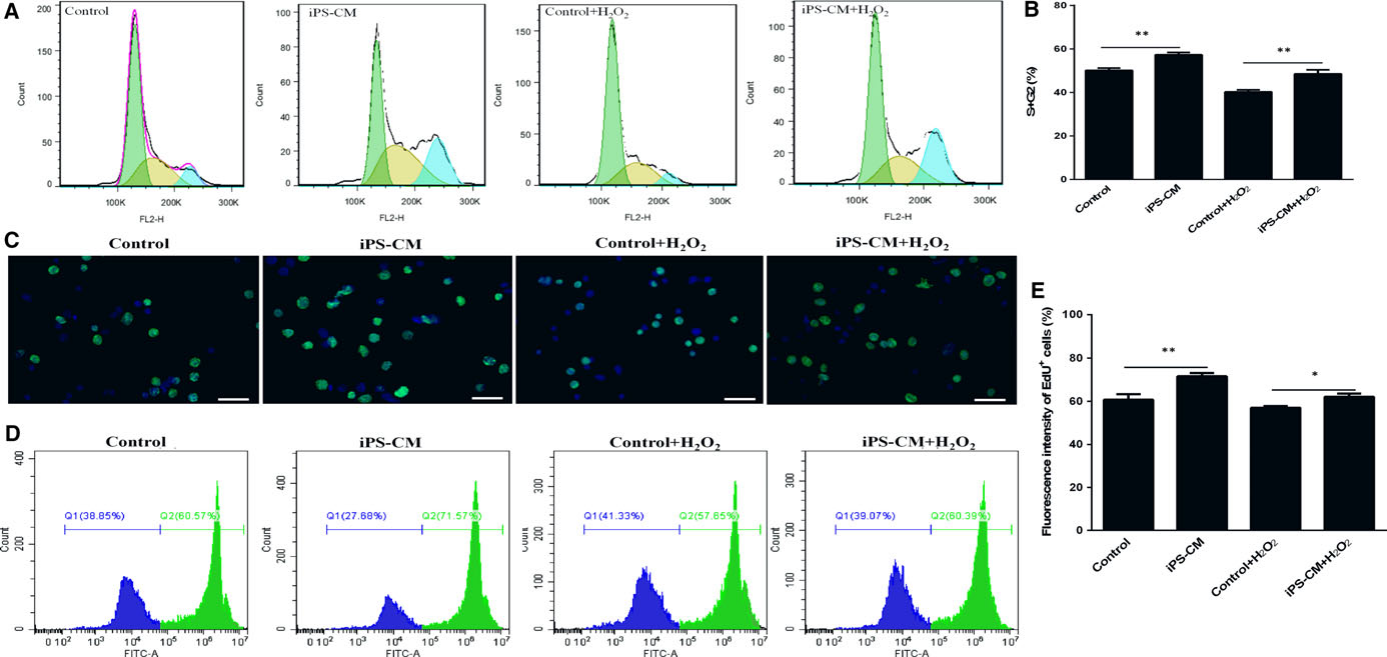
iPS‐CM promoted the proliferation viability of H_2_O_2_‐induced immature Leydig cells (ILCs). (A) The cell cycle assay was conducted to analyse the proliferation viability in different groups. (B) Quantification of the cell cycle distribution. (C) EdU staining images at an inverted fluorescence microscope. (D) Fluorescent density of EdU‐positive cells was detected by flow cytometry assays. (E) Quantification of the EdU‐positive cells. Mean ± SE, n = 3. *P <* *.05, **P <* *.01 designate significant differences. *Scale bars* 100 μm

The cell cycle assay was used to assess the effects of iPS‐CM on the proliferation of H_2_O_2_‐induced ILCs. Compared with control (ILCs in DMEM‐LG), H_2_O_2_ could inhibit cell proliferation, but iPS‐CM partly rescued the proliferation of H_2_O_2_‐induced ILCs (Figure 4A). The percentage of cell entering the S and G2 phases in control group was 50.43 ± 1.37%, which was less than that of iPS‐CM group (57.72 ± 1.45%) (**P <* *.01). Meanwhile, the percentage of cell entering the S and G2 phases in iPS‐CM+H_2_O_2_ group (50.56 ± 1.31%) was also more than that of control+H_2_O_2_ group (40.55 ± 1.12%) (**P <* *.01) (Figure 4B). In addition, EdU staining showed that the EdU‐positive cells in control group was less than that of iPS‐CM group, and iPS‐CM+H_2_O_2_ group was more than control+H_2_O_2_ group (Figure 4C). Meanwhile, the flow cytometry assays also demonstrated that the fluorescent density of EdU‐positive cells in control group was less than iPS‐CM group (**P <* *.01), and iPS‐CM+H_2_O_2_ group was more than that of control+H_2_O_2_ group (**P <* *.01) (Figure 4D,E). These results suggested that iPS‐CM could promote the proliferation of ILCs.

### 3.5 Effects of iPS‐CM on medium testosterone (T) levels and steroidogenic enzyme expressions of immature Leydig cells

**Figure 5:**
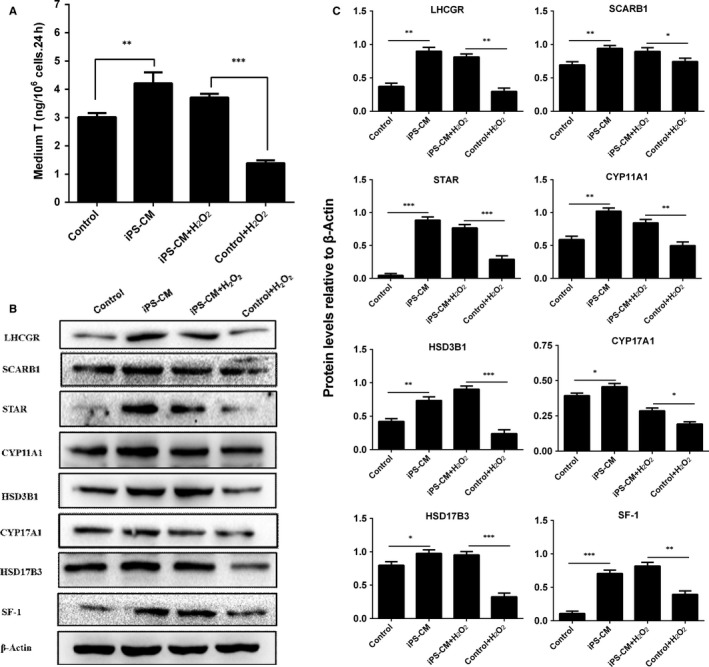
Medium testosterone (T) and protein expression levels of H_2_O_2_‐induced immature Leydig cells (ILCs) with or without iPS‐CM treatments. (A) Medium testosterone (T) levels in different groups. Leydig cell proteins: (B) Western blotting band in different groups, (C) quantification of protein levels. Mean ± SE, n = 3. *P <* *.05, **P <* *.01, ***P <* *.01 designate significant differences

To explore the effects of iPS‐CM administration on the testosterone synthesis of ILCs, the medium testosterone levels in different groups were detected by radioimmunoassay. The result showed that iPS‐CM could improve the medium testosterone levels compared with control (ILCs in DMEM‐LG) (**P <* *.01) and could also inhibit the decrease in the medium testosterone levels in H_2_O_2_‐induced ILCs (***P <* *.001) (Figure 5A). These data suggested that iPS‐CM could promote the testosterone synthesis of ILCs.

Western blotting was conducted to detect the steroidogenic enzyme expression levels of LHCGR, SCARB1, STAR, CYP11A1, HSD3B1, CYP17A1, HSD17B3 and SF‐1 in ILCs with different treatments (Figure 5B). Statistically, we found that iPS‐CM could significantly up‐regulate the expression levels of LHCGR, SCARB1, STAR, CYP11A1, HSD3B1, CYP17A1, HSD17B3 and SF‐1 compared with control. Meanwhile, iPS‐CM could also inhibit the down‐regulation of LHCGR, SCARB1, STAR, CYP11A1, HSD3B1, CYP17A1, HSD17B3 and SF‐1 in H_2_O_2_‐induced ILCs (Figure 5C). This result confirmed that iPS‐CM could promote the steroidogenic enzyme expressions of ILCs.

### 3.6 The potential mechanisms underlying the promotion anti‐apoptosis and proliferation of immature Leydig cell by iPS‐CM

**Figure 6:**
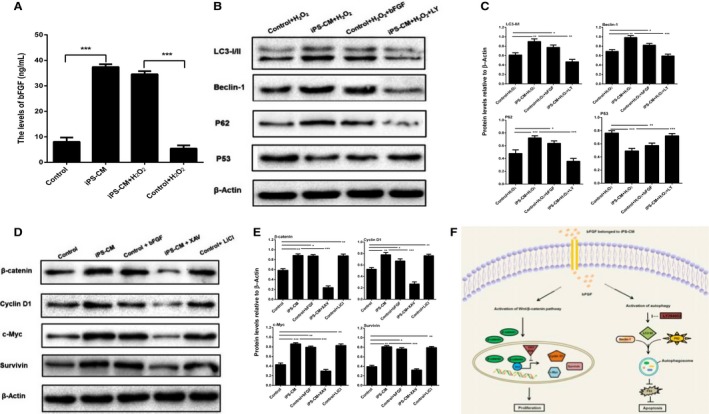
The potential mechanisms underlying the promotion of immature Leydig cell (ILC) anti‐apoptosis and proliferation by iPS‐CM. (A) Quantification of the bFGF levels of medium in different groups using ELISA. (B) Assessment of protein expression levels of LC3 I/II, Beclin‐1, P62 and P53 in different groups using Western blotting assays. (C) Quantification of Western blotting assays. (D) Assessment of protein expression levels of β‐catenin, cyclin D1, c‐Myc and survivin in different groups using Western blotting assays. (E) Quantification of Western blotting assays. (F) Diagram of the potential mechanisms underlying the promotion of ILC anti‐apoptosis and proliferation by iPS‐CM. Mean ± SE, n = 3. *P <* *.05, **P <* *.01, ***P <* *.01 designate significant differences

To understand the potential mechanisms underlying the promotion anti‐apoptosis and proliferation of ILCs by iPS‐CM, we firstly compared the levels of several special growth factors such as bFGF, IGF‐1 and VEGF in the medium of iPS‐CM group with those of control group (ILCs in DMEM‐LG) at the H_2_O_2_ induction or not by ELISA. There was just bFGF but not IGF‐1 and VEGF (data not shown) having significant difference. The level of bFGF in iPS‐CM was higher than that of DMEM‐LG with or without H_2_O_2_ treatment (***P <* *.001) (Figure 6A). Based on this result, exogenous bFGF (20 ng/mL) was added into the medium of control (DMEM‐LG) to establish a new group for mimicking the iPS‐CM group, and the underlying signalling pathways were further explored.

To elucidate the role of iPS‐CM in the activation of autophagy to suppress apoptosis of H_2_O_2_‐induced ILCs, the expression levels of autophagic proteins such as LC3‐I/II, Beclin‐1, P62, and apoptotic protein P53 among different groups were assessed by Western blotting (Figure 6B). The ratio of LC3 II/I and the levels of Beclin‐1 and P62 were significantly up‐regulated, but the expression of P53 was inhibited by iPS‐CM treatment in H_2_O_2_‐induced ILCs compared with the control. Additionally, exogenous bFGF added into DMEM‐LG could achieve the similar effects of iPS‐CM. However, the up‐regulated autophagic proteins and the down‐regulated apoptotic protein were also inhibited in iPS‐CM+H_2_O_2_ by LY294002 (LY, 25 μmol/L) (Figure 6C), an inhibitor of autophagy.^38^ These results suggested that iPS‐CM might markedly depend on inherent bFGF to suppress the apoptosis of ILCs through activation of autophagy pathway.

The Wnt/β‐catenin signalling pathway is the classic pathway controlling cell proliferation.^22^ The results of Western blotting analysis showed that the levels of β‐catenin and its target proteins cyclin D1, c‐Myc and survivin in iPS‐CM group were higher than those in control group (ILCs in DMEM‐LG). At the same time, all proteins could be up‐regulated by exogenous bFGF added into DMEM‐LG. Furthermore, those proteins could also be up‐regulated by LiCl (20 mmol/L) added into DMEM‐LG, which is a widely used in the activation of Wnt/β‐catenin signalling.^39^, ^40^ However, those proteins were again down‐regulated in iPS‐CM by XAV939 (XAV, 10 μmol/L) (Figure 6D,E), an inhibitor of the Wnt/β‐catenin signalling pathway.^41^ These results indicated that iPS‐CM might be mainly relied on inherent bFGF to promote the proliferation of ILCs through Wnt/β‐catenin signalling pathway. And the diagram of the mechanisms underlying the promotion anti‐apoptosis and proliferation of ILCs by iPS‐CM is shown in Figure 6F.

## 4 DISCUSSION

Male hypogonadism is characterized by the low production of testosterone, which is associated with typical symptoms including mood disturbance, sexual dysfunction, decreased muscle mass and strength, and decreased bone mineral density.^42^ Currently, testosterone replacement therapy is applied to treat androgen‐deficient males with primary Leydig cell failure and can achieve some alleviation of symptoms. However, the therapy is limited by the risks of side effects, such as reducing the rate of spermatogenesis, increasing cardiovascular and prostate complications.^43^ Leydig cell transplantation may be a better alternative in providing physiological patterns of hormone for a longer period of time.^44^ In this study, we systematically investigated iPS‐CM could promote viabilities including proliferation and anti‐apoptosis, and testosterone synthesis of immature Leydig cell (ILCs), which would be better used for future clinical cell transplantation.

iPSCs cultured in vitro could secrete many cytokines, chemokines, growth factors, metabolites and bioactive lipids into the medium. A wide range of products from iPSC secretion could reduce apoptosis, oxidative stress and fibrosis, as well as improve cardiac function in diabetic model of rats.^45^ Apoptosis, as a basic character of cells, is a physiological process of cell death that plays a key role in a variety of biologic systems.^46^, ^47^ H_2_O_2_ is very ideal inductor for establishment apoptosis model. In this study, ILCs were exposed to different concentrations of H_2_O_2_, and we scan out the optimal dose of 200 μmol/L for the apoptosis model. The mechanisms for apoptosis contain direct damage to the mitochondria by ROS and indirect mitochondrial depolarization by apoptotic‐related BCL‐2 family proteins.^48^ BAX, a pro‐apoptotic and pore‐forming cytoplasmic protein in the BCL‐2 family, is translocated to the outer mitochondrial membrane and affects permeability from the intermembrane space into the cytosol, which later causes cell death.^49^ Conversely, BCL‐2, an anti‐apoptotic protein in the BCL‐2 family, lies in the cytoplasm of the outer mitochondrial membrane, nuclear envelope and endoplasmic reticulum.^50^, ^51^ It was demonstrated that BCL‐2 in fibroblasts was closely related to mitochondrial homeostasis and cell viability.^52^ In this study, H_2_O_2_‐treated ILCs showed an increase in intracellular ROS, a loss of ▵Ψm, up‐regulation in the expression of BAX and down‐regulation in the expression of BCL‐2. However, the generation of ROS was inhibited by treatment with iPS‐CM. We demonstrated that iPS‐CM protected ILCs against H_2_O_2_‐induced apoptosis by stabilizing ▵Ψm and decreasing the expression of BAX, and increasing the expression of BCL‐2. These results indicated that iPS‐CM had protective effects of H_2_O_2_‐induced ILCs. Neel and Singla also found that iPS‐CM could decrease the number of cardiac apoptosis in the streptozotocin of diabetic cardiomyopathy (SIDC) rat model.^21^

Zhang et al^20^ reported that iPS‐CM could not only suppress apoptosis by inhibiting p53/p21 and p16/pRb pathways, but also promote proliferation by attenuating G1 phase arrest of cell cycle in H9C2 cells. In our study, we also found that iPS‐CM could increase the percentage of ILCs entering the S and G2 phases and EdU staining‐positive ILCs with or without H_2_O_2_ treatment, which indicated that iPS‐CM could significantly promote the proliferation of ILCs. iPS‐CM could enhance alveolar epithelial regeneration in vivo partially due to containing hepatocyte growth factor.^53^ iPS‐CM could also promote the growth of other cells.^54^, ^55^

In addition, we also found that iPS‐CM could significantly increase the medium testosterone levels of ILCs with or without H_2_O_2_ treatments. The testosterone homeostasis in rat ILCs mainly depends on the cholesterol membrane receptor and transporters such as LHCGR, SCARB1 and STAR, and the testosterone biosynthetic enzymes such as CYP11A1, HSD3B1, CYP17A1 and HSD17B3. The results of Western blotting showed that iPS‐CM could significantly increase the expression levels of membrane receptor LHCGR, cholesterol transporters SCARB1 and STAR, and testosterone biosynthetic enzymes CYP11A1, HSD3B1, CYP17A1 and HSD17B3, which could be contributed to the improvement of testosterone levels. Their increased levels were consistent with SF‐1 up‐regulation, suggesting that iPS‐CM up‐regulated these proteins via increasing SF‐1 expression. Indeed, many studies had showed that CYP11A1, HSD3B1, CYP17A1 and HSD17B3 promoters had SF‐1 binding sites.^56^, ^57^ Although the exact mechanism is still unclear, the significant increase in SF‐1, a critical transcription factor for the expression of steroidogenic enzymes,^7^ might be involved in possible inherent mechanism.

As is known to all that the activation of autophagy can protect cells against apoptosis and inflammation,^31^, ^32^ in this study, it was discovered that the level of bFGF in iPS‐CM was higher than that of control (DMEM‐LG) with or without H_2_O_2_ treatment. iPS‐CM could up‐regulate the expression of autophagic proteins LC3 II/I, Beclin‐1 and P62 but down‐regulate the expression of apoptotic protein P53 in H_2_O_2_‐induced ILCs. However, the up‐regulated autophagic proteins and the down‐regulated apoptotic protein could be inhibited by LY294002 (25 μmol/L), an inhibitor of autophagy.^38^ Additionally, exogenous bFGF (20 ng/mL) added into DMEM‐LG could achieve the similar effects of iPS‐CM. These results suggested that iPS‐CM might act as an agonist of autophagy pathway to inhibit apoptosis of ILCs, which was markedly contributed to the inherent bFGF. The Wnt/β‐catenin signalling pathway is the classic pathway‐mediated cell proliferation.^22^ A closely related report demonstrated that XAV939 (an inhibitor of the Wnt/β‐catenin signalling pathway) could counteract the proliferation of preterm umbilical cord mesenchymal stem cells (UC‐MSCs) compared to term UC‐MSCs.^58^ In this study, it was showed that iPS‐CM could significantly promote the expression levels of Wnt/β‐catenin signalling proteins such as β‐catenin, cyclin D1, c‐Myc and survivin, but could be inhibited by XAV939 (10 μmol/L). Exogenous bFGF (20 ng/mL) or LiCl (20 mmol/L) added into DMEM‐LG could obtain almost similar effects of iPS‐CM. LiCl is a widely used GSK‐3β inhibitor that results in the activation of Wnt/β‐catenin signalling pathway.^59^ These data suggested that iPS‐CM might mainly rely on inherent bFGF to promote the proliferation of ILCs through up‐regulation of Wnt/β‐catenin signalling pathway. Taken as a whole, our study discovered that both the inhibition ILC apoptosis mediated by the activation of autophagy pathway and the promotion ILC proliferation mediated by the Wnt/β‐catenin signalling pathway might be attributed to the inherent bFGF of iPS‐CM. While this study just hints the potential mechanisms underlying the promotion anti‐apoptosis and proliferation of ILCs by iPS‐CM, the convincing molecular mechanisms should need further study in the future.

In conclusion, we reported that iPS‐CM might mainly rely on inherent bFGF to dramatically inhibit H_2_O_2_‐induced apoptosis, stimulate proliferation and enhance testosterone production of ILCs. The anti‐apoptotic role of iPS‐CM was correlated with the down‐regulation of BAX and P53 as well as up‐regulation of BCL‐2. The promotion proliferation role of iPS‐CM was involved in the up‐regulation of β‐catenin, cyclin D1, c‐Myc and survivin. Meanwhile, iPS‐CM could promote the testosterone production through up‐regulation of the steroidogenic enzyme expressions, which might be due to the increase of SF‐1 expression. Additionally, the potential mechanisms including activation of autophagy pathway participated in ILC anti‐apoptotic and the Wnt/β‐catenin signalling pathway involved in promotion ILC proliferation after iPS‐CM treatment were illuminated. This study had developed a new way to improve the viabilities and function of ILCs for future clinical cell transplantation.

## COMPETING INTERESTS

The authors have declared that no competing interests exist.
